# Complete genome sequence of *Lacticaseibacillus rhamnosus* strain VHProbi F20, isolated from a healthy adult

**DOI:** 10.1128/MRA.00987-23

**Published:** 2023-11-20

**Authors:** Chaoqun Guo, Hongchang Cui, Qian Wang, Zhi Duan

**Affiliations:** 1 Qingdao Vland Biotech Group Co, Qingdao, China; Department of Biological Sciences, Wellesley College, Wellesley, Massachusetts, USA

**Keywords:** complete genome sequence, *Lacticaseibacillus rhamnosus*, VHProbi F20

## Abstract

*Lacticaseibacillus rhamnosus* strain VHProbi F20 is a strain isolated as part of a search for probiotics to prevent and help fight against respiratory tract infection caused by *Haemophilus influenzae*. Here, we investigate the whole-genome sequence of this strain. The whole genome contains a chromosome and a plasmid.

## ANNOUNCEMENT

Fecal samples were collected from healthy adults in a rural area of Tibet (91.1°E, 29.6°N) on 12 September 2021. All procedures were in accordance with the Declaration of Helsinki. These samples were serially diluted with 0.85% NaCl solution, and the 10^−6^ dilution was spread onto De Man-Rogosa-Sharpe agar plates and cultured at 37°C for 36 h to enrich bacteria. The strain VHProbi F20 was classified as *Lacticaseibacillus rhamnosus* using 16S rRNA sequencing; primers 27F (5′-AGAGTTTGATCCTGGCTCA-3′) and 1492R (5′-GGTTACCTTGTTACGACTT-3′) were used (1). The Illumina HiSeq 2500 and the PacBio RS II Single Molecule Real Time (SMRT) sequencing platform were combined to sequence the genomes of this strain. A single colony from the enrichment plate was cultured for DNA extraction using the same culture conditions as for enrichment. Total DNA was extracted using the Genomic DNA Purification Kit (Promega, USA) following its instruction. The Illumina library and the PacBio library used the same DNA samples. Template DNA was sheared into 400 bp using a Covaris M220 focused acoustic shearer for the Illumina library creation using the TruSeq library preparation kit from Illumina. The prepared libraries were then used for paired-end Illumina sequencing (2 × 150 bp). For the PacBio sequencing, DNA was spun in a Covaris G-Tube (Covaris, MA, USA) to be sheared into fragments. Then, ~10-kb fragments were purified, end-repaired, and ligated with the SMRTbell sequencing adapters following the PacBio’s recommendation. Next, a ~10-kb insert library was prepared using SMRTbell Express Template Prep Kit v.2.0 and sequenced on one SMRT cell using standard methods. Finally, 8,619,814 raw reads and 8,532,886 clean reads were generated using the Illumina sequencing platform to reach a depth of 972.54-fold coverage and 291,281 raw reads (*N*_50_, 229,970 bp) were generated using the PacBio sequencing platform. Adapters, non-A, G, C, T bases of the 5′-end reads containing >10% unknown *N* and low-quality reads were removed by using Sickle v.1.33 (https://github.com/najoshi/sickle). The PacBio raw reads were then assembled into a scaffold using hierarchical genome assembly process (HGAP v.4.0) (2) and Canu v.2.2 (3). Error correction of the PacBio assembly results was performed using the Illumina reads by Pilon v.1.22 (4). If there was an overlap at both ends of the scaffold, one end of the overlap was cut off and the sequence was looped to a circular sequence. A complete circular genome with a chromosome and a plasmid was obtained by the final assembly using Unicycler v.0.4.8 (5). CheckM v.1.1.3 was used to check the quality of the assembled genome sequence with the completeness of 99.46%, contamination of 0%, and strain heterogeneity of 0% (6). Genome annotations were conducted using the NCBI Prokaryotic Genome Annotation Pipeline v.6.1 (7). Default parameters were used for all software unless otherwise specified.

The genome of *L. rhamnosus* VHProbi F20 contains a 3,006,636-bp circular chromosome and a 14,130-bp circular plasmid with GC contents of 46.69%. There were 2,712 protein-coding genes, 15 rRNA genes (5S rRNA, 16S rRNA, and 23S rRNA), 59 tRNA genes, 3 ncRNA genes, and 46 pseudogenes in the genome.

## Data Availability

The 16S rRNA sequence and complete genome sequence of *Lacticaseibacillus rhamnosus* VHProbi F20 have been deposited at GenBank with accession numbers OQ592685 and CP094952 to CP094953, SRA accession numbers SRR24831249 and SRR24831250, BioProject accession number PRJNA821980, and BioSample accession number SAMN27162334.
